# Signalling pathways in UHRF1-dependent regulation of tumor suppressor genes in cancer

**DOI:** 10.1186/s13046-016-0453-5

**Published:** 2016-11-14

**Authors:** Mahmoud Alhosin, Ziad Omran, Mazin A. Zamzami, Abdulrahman L. Al-Malki, Hani Choudhry, Marc Mousli, Christian Bronner

**Affiliations:** 1 Department of Biochemistry, Faculty of Science, King Abdulaziz University, Jeddah, Saudi Arabia; 2 Cancer Metabolism and Epigenetic Unit, Faculty of Science, King Abdulaziz University, Jeddah, Saudi Arabia; 3 Cancer and Mutagenesis Unit, King Fahd Medical Research Center, King Abdulaziz University, Jeddah, Saudi Arabia; 4 Center of Innovation in Personalized Medicine, King Abdulaziz University, Jeddah, Saudi Arabia; 5 College of Pharmacy, Umm Al-Qura University, 21955 Makkah, Kingdom of Saudi Arabia; 6 Laboratoire de Biophotonique et Pharmacologie, UMR 7213 CNRS, Université de Strasbourg, Faculté de pharmacie, 74 route du Rhin, 67401 Illkirch, France; 7 Institut de Génétique et de Biologie Moléculaire et Cellulaire (IGBMC), INSERM U964 CNRS UMR 7104, Université de Strasbourg, 1 rue Laurent Fries, 67404 Illkirch, France; 8 Biochemistry Department, Faculty of Sciences, Cancer and Mutagenesis Unit, King Fahd Centre for Medical Research, King Abdulaziz University, P. O. Box 80203, Jeddah, 21589 Saudi Arabia

**Keywords:** Epigenetic, DNA methylation, p16^INK4A^, p53, p73, Tumor suppressor genes, UHRF1

## Abstract

Epigenetic silencing of tumor suppressor genes (TSGs) through DNA methylation and histone changes is a main hallmark of cancer. **U**biquitin-like with P**H**D and **R**ING **F**inger domains **1** (UHRF1) is a potent oncogene overexpressed in various solid and haematological tumors and its high expression levels are associated with decreased expression of several TSGs including *p16* ^*INK4A*^, *BRCA1*, *PPARG* and *KiSS1*. Using its several functional domains, UHRF1 creates a strong coordinated dialogue between DNA methylation and histone post-translation modification changes causing the epigenetic silencing of TSGs which allows cancer cells to escape apoptosis. To ensure the silencing of TSGs during cell division, UHRF1 recruits several enzymes including histone deacetylase 1 (HDAC1), DNA methyltransferase 1 (DNMT1) and histone lysine methyltransferases G9a and Suv39H1 to the right place at the right moment. Several in vitro and in vivo works have reported the direct implication of the epigenetic player UHRF1 in tumorigenesis through the repression of TSGs expression and suggested UHRF1 as a promising target for cancer treatment. This review describes the molecular mechanisms underlying UHRF1 regulation in cancer and discusses its importance as a therapeutic target to induce the reactivation of TSGs and subsequent apoptosis.

## Background

**Fig. 1:**
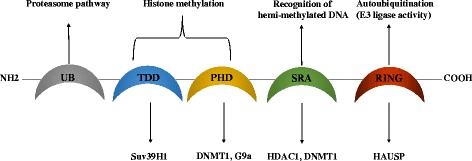
Schematic representation of UHRF1 structure and its role in the regulation of epigenetic code. Abbreviation: UBL (ubiquitin-like) domain, TTD (Tandem Tudor Domain), PHD (Plant Homeo Domain) domain, SRA (Set and Ring Associated) domain and RING (Really Interesting New Gene) domain. RING domain has an E3 ligase activity involved in UHRF1 autoubiquitination. UHRF1 is protected from this process by its interaction with herpes virus-associated ubiquitin-specific protease (HAUSP). Through its SRA domain, UHRF1 recognizes hemi-methylated DNA during DNA replication and interacts with DNA methyltransferase 1 (DNMT1) and histone deacetylase 1 (HDAC1). UHRF1 can also interact with DNMT1 via its PHD domain. Both PHD and TTD are involved in the readout of histone methylation that are catalysed by histone methyltransferases G9a and Suv39H1. The UBL domain could be involved in the proteasome pathway

Beside genetic alterations in cancer cells, epigenetic changes (DNA methylation and histone modifications) can also induce silencing of tumor suppressor genes allowing cancer cells to escape apoptosis and promote tumor progression [1–4]. The epigenetic reader UHRF1 **(U**biquitin-like, containing P**H**D and **R**ING **F**inger domains **1)**, an oncogene overexpressed in various human cancer cells is one of the major players involved in apoptosis inhibition by inducing epigenetic silencing of TSGs [5–8]. UHRF1 has several functional domains (Fig. 1): UBL (ubiquitin-like) domain, TTD (Tandem Tudor Domain), PHD (Plant Homeo Domain) domain, SRA (Set and Ring Associated) domain and RING (Really Interesting New Gene) domain. Through these domains, UHRF1 interacts with various proteins, forming a large macro-molecular protein complex called ECREM « Epigenetic Code Replication Machinery », which is engaged in the transmission of the epigenetic code including the silencing of TSGs, from a mother cancer cell to daughter cells during cell proliferation [5, 6]. By its original structure, UHRF1 might be the driver of this complex to ensure the replication of the epigenetic code (DNA methylation and histone code) after DNA replication, allowing cancer cells to conserve the silencing of TSGs during cell division. The SRA domain of UHRF1 behaves as a “hand” with two fingers that serve to flip out the methylated cytosine with subsequent recruitment of DNMT1 to methylate the cytosine of the newly synthetized DNA strand [9–11]. This recruitment was proposed to be under the control of SRA binding to hemi-methylated DNA, challenging enhanced activity of the UHRF1 RING finger that exhibits E3 ligase activity towards histone H3 [12, 13]. The TTD exhibits affinity for methylated histones and allows to confer a fabulous property to UHRF1 of connecting DNA methylation to histone modifications [14, 15]. Recently, new insights have been gained into the mechanism of this connection. Indeed, Fang et al., showed how UHRF1 can coordinately recognize histone modifications and hemi-methylated DNA [16]. UHRF1 adopts a closed conformation, in which a spacer located in the C-terminal region of UHRF1 binds to the TTD and thus hinders this latter from H3K9me3 binding [16]. The SRA domain binds to the PHD and inhibits this latter from H3R2 recognition. In the presence of hemi-methylated DNA, the intramolecular interactions were impaired thanks to a preferred affinity for hemi-methylated DNA vs PHD domain. Subsequently, H3K9me3 recognition by TTD–PHD is facilitated and thus is supporting a crucial role for UHRF1 in connecting DNA methylation with histone post-translational modifications. The close conformation has been recently proposed as being regulated by phosphatidyl-5-phosphate [17], a small molecule involved in cell signaling and cell traffic [18]. The authors suggested that phosphatidyl-5-phosphate, since its concentration is varying from G1 to S phase, might determine the localization of UHRF1 in chromatin during the cell cycle [17]. Consistently with these studies, the contribution of UHRF1 to the interconnection between DNA methylation and histone methylation has been further deciphered by a new study, which supports a model in which H3K9 methylation recognition, through the TTD domain, while not essential, promotes DNA methylation maintenance [19].

Recently, new highly interesting functions were uncovered for UHRF1. One of the most interesting is a sensor role for interstrand crosslinks, showing that UHRF1 is also involved in DNA repair processes [20–22]. Of note, it was also shown that the decrease of UHRF1 protein levels is a major cause of DNA demethylation in embryonic stems cells [23]. Therefore, UHRF1 appears to have a triple role during cell proliferation, *i.e.,* in DNA methylation pattern inheritance, sensor of DNA crosslinks and a facilitator of DNA demethylation during development. It is thus easily conceivable that a dysregulation of one or more of these functions may lead to genomic alteration and thus to cancer.

Considering the fact that UHRF1 is overexpressed in various solid [5, 24] and haematological tumors [25, 26] and that UHRF1 via its domains (Fig. 1) guarantees a strong relationship between DNA methylation and histone post-translational changes [5, 6, 27, 28], targeting this epigenetic actor could be a new promising anticancer strategy. In this review, we highlight the role of UHRF1 in the epigenetic silencing of TSGs and the molecular mechanisms underlying UHRF1 regulation in cancer cells as well as the increasing importance of UHRF1 as a promising target for anticancer therapy.

## Role of UHRF1 in the epigenetic silencing of TSGs in cancer

Several TSGs, among which *p16* ^*INK4A*^ seems to be the most interesting, were shown as being silenced through UHRF1-mediated epigenetic modifications, mainly DNA methylation [6, 29]. The tumor suppressor gene *p16* ^*INK4A*^ is involved in the G1/S cell cycle checkpoint and its lost expression leads to apoptosis inhibition, enhanced cell proliferation and loss of cell contact inhibition. UHRF1 uses its functional domains to exert epigenetic inhibitory effects on TSGs including *p16* ^*INK4A*^ [30–32]. Indeed, one of the most important features of UHRF1’s structure, is the presence of an intriguing “Set and Ring Associated” domain (SRA), which is found only in the UHRF family [5]. Using its SRA domain, UHRF1 interacts with histone deacetylase 1 (HDAC1) and DNMT1 [7, 31, 33]. This interaction takes place at methylated promoter regions of several TSGs including *p16* ^*INK4A*^ *, p14* ^*ARF*^ (known as *p19* ^*ARF*^ in mouse), both encoded by the *CDKN2A gene* [34], and *RARalpha* [7]. However, to our knowledge no data are so far available in the literature about the consequence on p14^ARF^ and RAR protein levels [7]. Interestingly, UHRF1 depletion resulted in DNMT1 downregulation and an upregulation of *p16* ^*INK4A*^ [31]. In the same context, we have shown that the natural anti-cancer drug, epigallocatechin-3-gallate (EGCG) induces a significant decrease in UHRF1 and DNMT1 expression in Jurkat cells in association with p16^INK4A^ upregulation, cell cycle G1/S arrest and apoptosis [32]. The EGCG-induced *p16* ^*INK4A*^ upregulation was related to a significant decrease in UHRF1 protein binding to *p16* ^*INK4A*^ promoter [32]. Interestingly, wild type UHRF1 overexpression, but not SRA UHRF1 mutants, was able to decrease *p16* ^*INK4A*^ expression indicating that UHRF1 negatively controls the expression of *p16* ^*INK4A*^ in leukemia cells [32]. It appears that *p16* ^*INK4A*^ upregulation through a UHRF1 downregulation is a key mechanism of many natural drugs exhibiting anti-cancer properties [9, 29, 32].

UHRF1 was also shown to be overexpressed in colorectal cancer (CRC) and its overexpression is associated with CRC progression [35]. In this type of cancer, UHRF1 knockdown induced an upregulation of *p16* ^*INK4A*^, inhibition of cell proliferation and metastasis as well as apoptosis [35]. UHRF1 was also shown to be overexpressed in primary non-small cell lung cancer (NSCLC) and its high expression level was associated with an increase in the expression of DNMT1, DNMT3A, and DNMT3B and correlated with hypermethylation of *p16* ^*INK4A*^ promoter [36]. In accordance with this, enhanced UHRF1 expression was also reported in gastric cancer (GC), and correlated with tumor progression [37]. Again, UHRF1 depletion induced the reactivation of several TSGs, including *p16* ^*INK4A*^, and led to cell proliferation inhibition [37]. Recently, we showed that activation of CD47 in two human astrocytoma cell lines, upregulated the expression of UHRF1 with subsequent downregulation of *p16* ^*INK4A*^ [38]. All these studies support the existence of a common mechanism in cancer that UHRF1 regulates the expression of *p16* ^*INK4A*^ with subsequent inhibition of the apoptotic pathways. It is also noteworthy that UHRF1 regulates a plethora of other TSGs among which *RB1* especially in Jurkat and osteosarcoma cells [31, 39, 40], *CDX2, CDKN2A, RUNX3, FOXO4, PPARG, BRCA1* and *PLM,* in gastric cancer [37], *SOCS3* and *3OST2* in endometrial carcinoma [41] as well as *BRCA1* in cancer breast cell lines [42].

The overall well admitted mechanism of tumor suppressor gene silencing is thought to be DNA methylation as almost all promoters of TGS regulated by UHRF1 are hypermethylated. Note that UHRF1 is also able to silence, in DNA methylation dependent process, KiSS1, a gene known to have anti-metastasis functions [43]. However, it has not to be neglected that other mechanisms might be involved such as histone post-translational modifications. Indeed, considering that UHRF1 has several histone modifyers as partners, all these may putatively exert a contribution in the definitive interlocking of TSGs. For instance, UHRF1 has been shown to recruit histone lysine methyltransferase G9a to the BRCA1 promoter and with subsequent histone 3 lysine 9 methylation [42]. In another study, it has been reported that UHRF1 associates with PRMT5 (Protein arginine N-methyltransferase 5) in endometrial carcinoma [44]. In the same study, it has been shown that the promoters of TSGs *CH13* and *SHP1* were hypermethylated but whether there is a link between the activity of PRMT5 and TSGs silencing still remains elusive. But if it is the case, it would mean that UHRF1, by recruiting PRMT5 to the TSGs promoters, favors the participation of the dimethylation of arginine 8 of histone H3 (H3R8me2) and arginine 3 of histone H4 (H4R3me2) to gene silencing of TSGs *ST7* and *RBL2* [45]. Whilst these possibilities cannot and should not be discounted, it is worth pointing out that the complexity of UHRF1-dependent TSGs regulation might be as directly proportional to the size of the macromolecular UHRF1 complex.

One important member of this macromolecular complex is USP7 (Ubiquitin Specific Peptidase 7) or HAUSP (Herpes virus-Associated Ubiquitin-Specific Protease). HAUSP has been reported to regulate several TSGs, including *p53* [46]. The deubiquitinase HAUSP was shown to interact with UHRF1 to maintain its deubiquitination status protecting it from autoubiquitination and degradation by the proteasome [47–49]. Overexpression of HAUSP increased UHRF1 level while HAUSP downregulation induced UHRF1 ubiquitination causing its degradation via a proteasome-dependent process [48]. These findings indicate that HAUSP acts as a UHRF1 protector from autoubiquitination-mediated degradation using RING domain [48]. Recently, it has been shown that HAUSP controls the stability of UHRF1 not only by maintaining its deubiquitination, but also by promoting its chromatin association [50]. Indeed, HAUSP was shown to reduce the E3 ligase activity (autoubiquitination) of wild-type UHRF1 but not in the UHRF1 K659E mutant, disturbing the UHRF1 domain involved in its interaction with HAUSP [50]. Interestingly, HAUSP interaction with UHRF1 facilitated its binding to the H3K9me3 and induced a significant increase in its association with chromatin in the cervical cancer cell line Hela-60 [50]. Taken together, these data show that HAUSP has a dual regulatory role of UHRF1, by protecting it from autoubiquitination (Fig. 1) and by facilitating its association to the chromatin through the readout of the histone code. Therefore, HAUSP tandem may control TSGs expression via UHRF1 mediated by a ubiquitination/deubiquitination balance and might be further considered as a key mechanism involved in controlling cell proliferation and apoptosis [51].

A regulatory influence on TSGs by UHRF1 might also be mediated independently of DNA methylation, for instance through an enzymatic partner. Indeed, it has been demonstrated that UHRF1 inhibits the interplay between Tip60 and p53 [52]. Tip60, an essential partner of UHRF1 [53], acetylates p53 at K120 to induce apoptosis [54]. It was suggested that increased expression of UHRF1 found in cancer might be responsible for decreased activity of p53 and apoptosis failure in tumors [52].

All these findings indicate that UHRF1 is a main key in the epigenetic silencing of various TSGs in cancer. So, understanding the molecular mechanisms underlying UHRF1 overexpression in cancer will help to find new targets to inhibit UHRF1 expression which will allow cancer cells to undergo apoptosis through the reactivation of silenced TSGs. In other words, the goal would be to re-express TSGs in cancer cells to allow them to commit “suicide” via a re-activation of the apoptotic pathways.

## Signalling pathways involved in UHRF1 regulation in cancer cells

### Role of TSGs in UHRF1 regulation

The tumor suppressor gene *p53* is involved in controlling cell cycle at G1/S transition ensuring a successful cell division [55, 56]. *p53* is silenced in 50 % of human cancers causing loss of cell cycle G1/S checkpoints which allows cancer cells to escape apoptosis [57–59]. In contrast to *p53*, *p73,* a *p53* functional and structural homolog [60], is rarely mutated in cancer [61]. UHRF1 has been shown to be targeted by TSGs such *p53* and *p73* [62–64] suggesting that UHRF1 overexpression observed in many human cancer could result from abnormal TSGs expression or from non-functional TSGs. We have shown that thymoquinone (TQ) triggers apoptosis in the p53-deficient Jurkat cell line through the activation of p73 [63]. Interestingly the depletion of p73 in TQ-treated cells prevented UHRF1 from TQ-induced degradation, indicating that p73 negatively controls the expression of UHRF1 [63]. In the same context, UHRF1 expression was shown to be decreased by p53 upregulation as a response to anticancer drugs-induced DNA damage [62]. Taken together, these findings suggest that UHRF1 expression levels observed in cancer could result from defects in the expression of some TSGs such as *p53* and *p73*. Thus, UHRF1 is regulated by TSGs expression but by a feed-back mechanism can also control the activity of TSGs.

### Regulation of UHRF1 by miRNA

microRNAs (miRNAs: 18–25 nucleotides) are considered as negative regulators for several genes at the post-transcriptional level [65, 66]. These small noncoding RNAs exert their action by binding to the 3′-untranslated region (3′-UTR) of their target mRNA resulting in degradation of mRNA from more than 60 % of human genes [67–69]. Depending on the cellular function of miRNAs targets, these molecules could be either an oncogene or a tumor suppressor gene [70]. Moreover, several studies revealed a strong relationship between many cancers and either mutations or abnormalities in miRNAs expression [70, 71]. In this context, it has been shown that miR-206 acts as a tumor suppressor able to inhibit the expression of both oncogenes *c-Met* and *Bcl2*, that are overexpressed in various cancers including lung cancer [72]. miR-720 expression was shown to be significantly reduced in acute myeloid leukemia (AML) patients compared to normal controls, while its overexpression induced an upregulation of tumor suppressor *p53* leading to cell proliferation inhibition and apoptosis [73].

Considering the fact that UHRF1 overexpression, observed in cancer, is associated with decreased expression levels of several miRNAs which act as tumor suppressor genes, it can be thus speculated that the large quantities of the UHRF1 produced in tumors might result from abnormalities in the expression of miRNA. In agreement with this, it has been shown that UHRF1 overexpression in GC results from a reduction in the expression of miR-146a and miR-146b, which are known to act as tumor suppressors in GC [74]. Interestingly, miR-146a/b overexpression significantly decreased UHRF1 expression by directly targeting its binding sites (3′-UTR) triggering DNA demethylation-dependent reactivation of some TSGs such as *RUNX3* [74]. Reduction in GC migration and in metastasis were the consequences [74]. In contrast, the downregulation of miR-146a/b induced an increase in UHRF1 expression, further and definitively confirming that miR-146a/b negatively regulates the expression of UHRF1 in GC [74].

miR-9 acts as tumor suppressor in CRC and its expression has been observed to be decreased in CRC compared to corresponding normal tissues [75, 76]. UHRF1 expression was shown to be more pronounced in human CRC tissue than matched normal tissues and its overexpression was linked to decreased expression levels of miR-9 and reduced survival rates of CRC patients [77]. Interestingly, transfection of CRC cells by pre-miR-9 induced a significantly decrease in UHRF1 expression indicating that pre-miR-9 negatively controls UHRF1 expression and that UHRF1 overexpression in CRC may result from a decrease in the expression of miR-9 [77].

The tumor suppressor, miR-193a-3p, has been reported to inhibit NSCLC progression but the molecular pathways through which this miRNA induces its inhibitory effects are largely unknown [78]. Nevertheless, it has been recently observed that miR-193a-3p repressed the metastasis of lung cancer cells by targeting several proteins highly expressed in NSCLC including UHRF1 [79] indicating that miR-193a-3p negatively modulates the expression of UHRF1 in NSCLC. In the same way, the tumor suppressor gene, miR-145-5p and 145-3p as well, were shown to down-regulate UHRF1 in bladder cancer, with subsequent apoptosis by targeting genes such as *BIRC5* and *CENPF* [80]. The regulatory mechanism involves a direct targeting of miRNA to the 3′-UTR of UHRF1 mRNA. Interestingly, in this study *UHRF1* was reported to be upregulated in bladder cancer clinical specimens and to promote anti-apoptotic effects through regulation of several oncogenic genes [80]. Finally, it was suggested that *UHRF1* might be a useful prognostic marker for survival of bladder cancer patients [80]. More recently UHRF1 has been shown to be regulated by miR-101 in renal cell carcinoma [81].

**Fig. 2:**
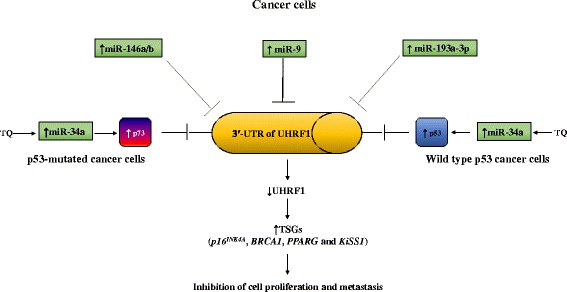
Schematic model of the role of miRNA in UHRF1 regulation in cancer cells. Several miRNAs act as tumor-suppressor by binding to the 3′-untranslated region (3′-UTR) of mRNA UHRF1 leading to its degradation. TQ increases the expression of miR-34a which leads to upregulation of p53 in wild type p53 cancer cells or p73 in p53-mutated cancer cells with subsequent UHRF1 inhibition. UHRF1 downregulation results in the reactivation of others TSGs including *p16* ^*INK4A*^, *BRCA1*, *PPARG* and *KiSS1* conducting to cell proliferation inhibition and apoptosis

miR-34a acts as a tumor suppressor in various cancers and its decreased expression levels were suggested to play a causal role in the initiation and progression of the tumor [82, 83]. Recently, it has been shown that TQ-encapsulated nanoparticles induce apoptosis in cancer cells by increasing the expression of miR-34a through p53-dependent pathway [84]. TQ, the most abundant biologically active component of black cumin oil, has potent anticancer activities on many human cancer cell lines by targeting numerous signalling pathways involved in the regulation of cell cycle and apoptosis including p53 and p73 pathways [29, 63, 85]. Considering that TQ targets UHRF1 in p53-mutated Jurkat cells through p73-dependent pathway [63] and that UHRF1 is also regulated by p53 [62], we might imagine that TQ decreases the expression of UHRF1 in cancer cells through the upregulation of miR-34a. Taken together, all these findings show that miRNAs exert a fine tuning of tumor-suppressor expression via regulation of UHRF1 expression (Fig. 2).

### Regulation of UHRF1 by CD47/ NF-ĸB pathway

Cancer cells use several strategies to escape immune system control. CD47, also called integrin-associated protein, is an immunoglobulin protein found on the surface of many human cells [86, 87]. Through its extracellular domain, CD47 exerts phagocytosis inhibitory activities via its ligation to the inhibitory receptor SIRPα (signal regulatory protein alpha) expressed on macrophages [88, 89]. Both solid and haematological tumors cells produce high amount of CD47 protein on their surface, compared with normal cells [90, 91]. CD47 overexpression is used by cancer cells to escape from the macrophages-mediated “don’t eat me” signal allowing tumor to progress [92–94]. Blocking the CD47/SIRPα axe-mediated “don’t eat me” signal allow macrophages to recognize CD47-positive cancer cells with subsequently destroy and elimination through a phagocytosis process [93, 95]. Several strategies have been used to target CD47 in cancer therapy. For instance, blocking CD47 function, using monoclonal antibodies against the CD47/SIRPα, showed in vitro and in vivo an important impact in many tumors overproducing CD47, such as leukemia and glioblastoma [94, 96]. It has been shown that CD47 is overexpressed in human melanoma and its depletion using CD47 siRNA significantly inhibited melanoma growth and metastasis [97]. In the same way, CD47 knockdown using specific siRNA inhibited the migration of intestinal epithelial cell by reducing the expression of cyclooxygenase-2 (COX-2) [98]. Recently, we showed that CD47 activation using 4 N1 (CD47 agonist) peptide induced a significant increase in the expression of both *UHRF1* gene and protein in human astrocytoma cell lines U87 and CCF-STTG1 (Grade IV) without affecting their expression in normal human astrocytes NHA [38]. The enhancement of UHRF1 expression induced by CD47 activation was associated with the phosphorylation of IĸBα, a NF-ĸB inhibitor, downregulation of *p16* ^*INK4A*^ and enhancement of cell proliferation [38]. In contrast, antagonizing CD47 function using monoclonal antibody (B6H12) induced downregulation of UHRF1 accompanied by dephosphorylation of IĸBα, an upregulation of *p16* ^*INK4A*^ and decreased cell proliferation [38]. Interestingly, CD47 knockdown using siRNA in U87 cell line induced a significant downregulation of UHRF1 indicating that CD47 positively controls the expression of UHRF1 in glioblastoma cells [38].

**Fig. 3:**
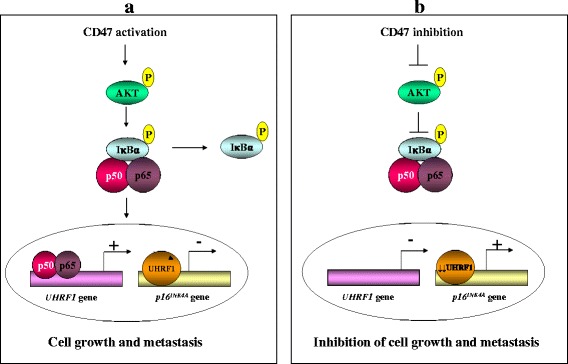
Role of CD47/NF-ĸB pathway in UHRF1 regulation. **a**. CD47 activation induces IĸBα phosphorylation allowing the translocation of the active NF-ĸB complex (p50 or p65) into nucleus to activate the *UHRF1* gene with subsequent *p16* ^*INK4A*^ repression and enhanced cell proliferation. **b**. Blocking CD47 function inhibits NF‐κB transactivation leading to decrease in binding of NF‐κB components (p50 or p65) to *UHRF1* promoter inducing cell proliferation inhibition via *p16* ^*INK4A*^ reactivation

The transcription factor NF-ĸB is activated in many human cancer including brain tumors [99, 100]. Considering that CD47-induced IĸBα phosphorylation was associated with UHRF1 upregulation and increased cell proliferation [38] and that CD47 activation induced the phosphorylation of Akt [101], we suggest that CD47 activation increases UHRF1 expression and promotes cell proliferation through the activation of the Akt-dependent NF-ĸB pathway (Fig. 3). Furthermore, we hypothesise that CD47 activation leads to IĸBα phosphorylation, thus releasing the active NF-ĸB complex (p50 and p65) which translocates into nucleus (Fig. 3a). p50 or p65 then binds to UHRF1 promoter inducing its activation and subsequently inhibits *p16* ^*INK4A*^ expression via UHRF1 binding to the promoter of this latter, promoting thus cell proliferation and metastasis (Fig. 3a). In contrast, CD47 function blocking will inhibit NF‐κB transactivation leading to decrease in NF‐κB binding to *UHRF1* promoter thereby inhibiting cell proliferation through *p16* ^*INK4A*^ reactivation (Fig. 3b). These findings indicate a key role of CD47 receptor in the regulation of UHRF1 expression most likely through the activation of the NF-ĸB pathway and also suggest that the overexpression of *UHRF1* observed in many human cancers might result from high levels of cell plasma membrane CD47.

### Regulation of UHRF1 by TRα1/Sp1 pathway

**Fig. 4:**
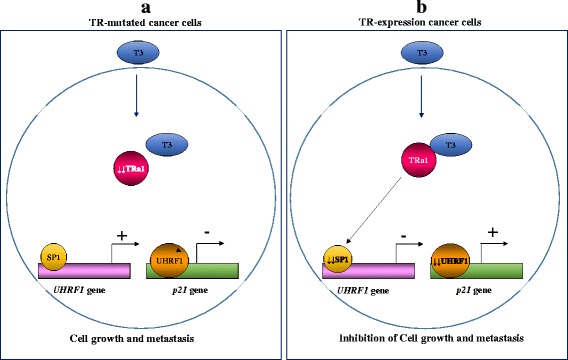
Schematic model of the role of TRα1/Sp1 pathway in the regulation of UHRF1. **a**. Abnormalities in T3/TRa1 pathway result in increasing of Sp1 binding to UHRF1 promoter causing its activation. UHRF1 overexpression suppresses the expression of *p21* gene with subsequent cell proliferation and metastasis. **b**. Exposure of TR-expressing cells to T3 induces a decrease in Sp1 binding to UHRF1 promoter causing its inactivation. UHRF1 repression results in p21 reactivation with subsequent inhibition of cell proliferation and metastasis

The thyroid hormone T3 (3,5,3′-triiodo-L-thyronine) is an important regulator of development, metabolism and cell proliferation [102]. The thyroid hormone receptors (TRs) act as tumor suppressors and their abnormal expression can lead to cancer progression [103]. T3 binds to TR regulating the expression of various genes including those involved in cell proliferation [103]. In this context, it has been shown that T3 negatively regulates the expression of UHRF1 in hepatoma cell line, which highly overexpresses TRα1 [104]. UHRF1 was shown to be overexpressed in liver cancer patients and its overexpression was accompanied with the size of tumor [104]. Exposure of TR-expressing HepG2 cells to T3 decreased the levels of UHRF1 mRNA and protein compared to TR-muted HepG2 [104]. T3-induced UHRF1 downregulation was associated with a decreased level of the transcription factor Sp1, upregulation of p21, G0/G1 cell cycle arrest and cell proliferation inhibition [104]. Interestingly, DNA ChIP assay showed that Sp1 binds to a specific site on UHRF1 promoter indicating that T3 regulates the expression of UHRF1 through the transcription factor Sp1 [104]. UHRF1 and Sp1 mRNA levels were also increased in hepatocellular carcinoma HCCs patient tissues compared to adjacent normal tissues in parallel with a decrease in the expression of TRα1 and p21 [104]. UHRF1 overexpression in HepG2 counteracted the T3-induced p21 overexpression, G0/G1 cell cycle arrest and cell proliferation inhibition allowing cell passage to G2/M phase [104]. Taken together, these findings show that T3/TRα1 pathway is involved in the regulation of UHRF1 expression in liver cancer through the transcription factor Sp1 (Fig. 4). This suggests that defects in T3/TR pathway in cancer cells result in UHRF1 overexpression through increasing of Sp1 binding to its promoter with subsequent cell proliferation and metastasis (Fig. 4a). Exposure of cancer cells to T3 induces a decrease in Sp1 binding to UHRF1 promoter causing its inactivation and subsequent p21 reactivation and cell proliferation inhibition (Fig. 4b).

### Inhibitors of UHRF1 and its signalling pathways

**Fig. 5:**
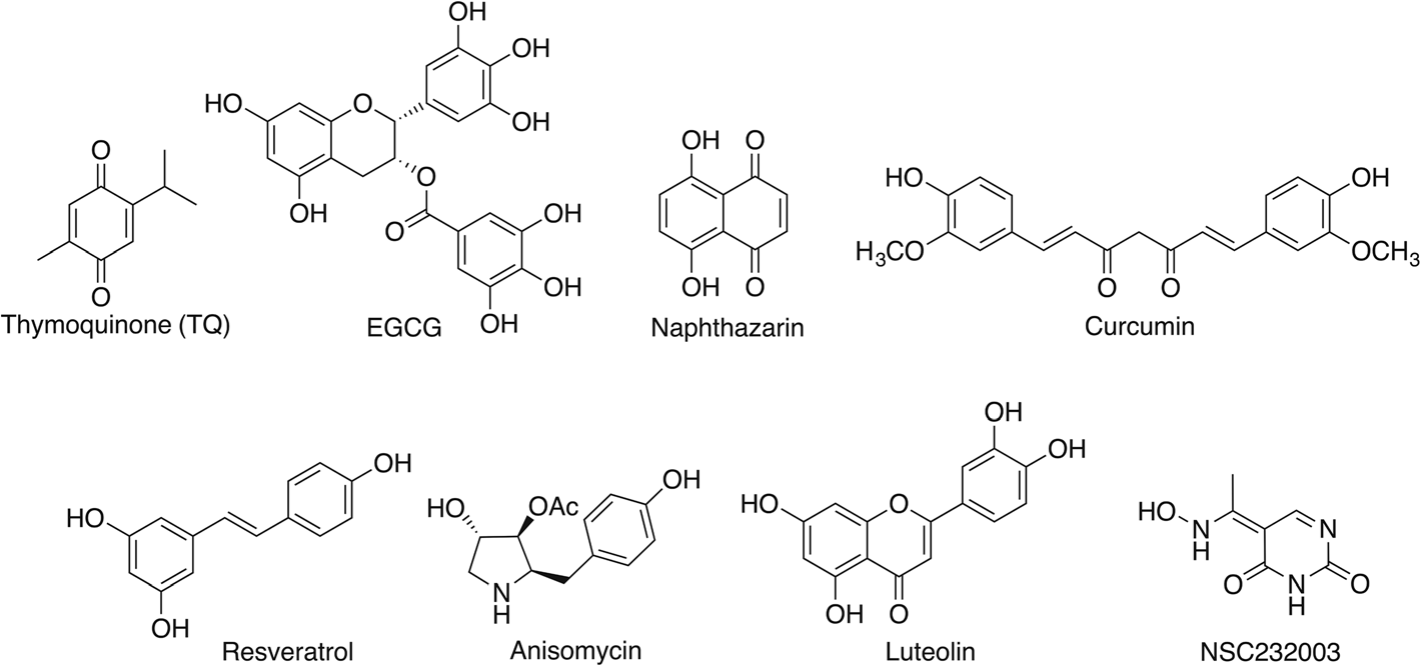
Chemical structure of UHRF1 inhibitor NSC232003 and of natural compounds targeting signaling pathways of UHRF1 expression

In vitro and in vivo studies have shown that a drug-induced inhibition of UHRF1 activity or expression leads to the reactivation of several tumor suppressor genes enabling cancer cells to undergo apoptosis [8, 29]. So far, only one direct inhibitor of UHRF1 has recently been reported [24]. Indeed, through a tandem virtual screening, a uracil derivative (NSC232003, Fig. 5), was described as a putative compound able to fit within the 5-methylcytosine binding pocket of the UHRF1 SRA domain. Interestingly, NSC232003 induces a global DNA hypomethylation probably through prevention of hemi-methylated DNA recognition by the SRA domain concomitantly to a disruption of UHRF1/DNMT1 interactions [24]. However, further investigations on this compound must be performed to check its capacity to reactivate silenced tumor suppressor genes through a UHRF1-dependent mechanism.

While, as stated above, the uracil derivative is the sole direct inhibitor, several inhibitors of the signaling pathways regulating UHRF1 expression are documented. UHRF1 expression was shown to be targeted by the natural product naphthazarin (Fig. 5) [105]. Naphthazarin induced cell proliferation inhibition and apoptosis of MCF-7 cells exposed to radiation through decreased binding of UHRF1, DNMT1 and HDAC1 to *p21* ^*CIP/WAF1*^ promoter [105]. In the same context, shikonin (Fig. 5), a natural naphthoquinone isolated from the Chinese traditional medicine Zi Cao (purple gromwell), has been shown to induce apoptosis in MCF-7 and HeLa cells, this effect was associated with a decrease in UHRF1 binding to *p16* ^*INK4A*^ promoter [106]. We have shown that TQ (Fig. 5) inhibits cell proliferation and induces apoptosis of Jurkat cells through p73 and caspase 3 upregulation and UHRF1 downregulation [63]. In accordance with these studies, we have also shown that treating B16F10 murine melanoma cells with curcumin induced a downregulation of UHRF1 and p73 upregulation, G1/S cell cycle arrest and apoptosis [107]. EGCG (Fig. 5) appears to take the same pathway to achieve the induction of apoptosis in Jurkat cells, *i.e.* UHRF1 downregulation and *p16* ^*INK4A*^ upregulation [32]. Although, several studies [37, 44, 108, 109] tend to show that reactivation of tumor suppressor gene involves a UHRF1 downregulation-dependent promoter demethylation, the contribution of other mechanisms are not excluded. Indeed, UHRF1 has been suggested to be a main player in the reactivation of the tumor suppressor gene *Pax1* (Paired box gene1) in several cancer cell lines in response to curcumin and resveratrol through a mechanism involving histone methylation and deacetylation rather than a DNA methylation-dependent process [110].

Other natural compounds, such as anisomycin and luteolin (Fig. 5), have been also reported to efficiently affect UHRF1 expression [111, 112]. Nevertheless, the mechanism of UHRF1 downregulation induced by natural compounds that target the signaling pathways of UHRF1 expression remains to be deciphered, but might involve the proteasome pathway. Indeed, for instance, the small molecule 17-AAG, a HSP90 inhibitor has been shown to induce UHRF1 ubiquitination leading to its degradation through proteasome-dependent pathway [113].

## Conclusion

The overexpression of the anti-apoptotic UHRF1 has been shown to coordinate the epigenetic silencing of several TSGs in many human haematological and solid cancers causing apoptosis inhibition. Via its structural domains, UHRF1 interacts with several proteins involved in the silencing of TSGs including DNMT1, HDAC1, G9a and Suv39H1 which make it a strong candidate to be the driver of this macro-protein complex to ensure the transmission of epigenetic code from a mother cancer cell to daughter cells during cell proliferation. The large quantities of UHRF1 produced in cancers could be result from abnormalities in the upstream regulatory mechanisms of UHRF1. This review highlighted the signalling pathways underlying UHRF1 regulation in cancer cells. Thus, understanding the molecular mechanisms involved in UHRF1 regulation will allow us to find new targets in order to inhibit the expression of UHRF1 allowing cancer cells to undergo apoptosis through a re-expression of tumor suppressor genes. As an interesting perspective in the field of cancer therapy, we have recently identified an inhibitor of UHRF1 (a uracil derivative) that targets the SRA domain with subsequent impact on DNMT1/UHRF1 interactions and decrease in global DNA methylation [24].

## Abbreviations

3′-UTR: 3′-untranslated region
CRC: Colorectal cancer
DNMT1: DNA methyltransferase 1
ECREM: Epigenetic Code Replication Machinery
EGCG: Epigallocatechin-3-gallate
GC: Gastric cancer
HAUSP: Herpes virus-Associated Ubiquitin-Specific Protease
HDAC1: Histone deacetylase 1
NSCLC: Nonsmall cell lung cancer
PDEs: Phosphodiesterases
PHD: Plant Homeo Domain
RING: Really Interesting New Gene domain
SRA: Set and Ring Associated domain
T3: Thyroid hormone 3
TQ: Thymoquinone
TRs: Thyroid hormone receptors
TSGs: Tumor suppressor genes
TTD: Tandem Tudor Domain
UBL: Ubiquitin-like domain
UHRF1: **U**biquitin-like with P**H**D and **R**ING **F**inger domains **1**
